# Blunt thoracic aortic injury and TEVAR: long-term outcomes and health-related quality of life

**DOI:** 10.1007/s00068-020-01432-y

**Published:** 2020-07-06

**Authors:** Dennis Hundersmarck, Quirine M. J. van der Vliet, Lotte M. Winterink, Luke P. H. Leenen, Joost A. van Herwaarden, Constantijn E. V. B. Hazenberg, Falco Hietbrink

**Affiliations:** 1 Department of Surgery, University Medical Center Utrecht, Heidelberglaan 100, Post-office 85500, 3508 GA Utrecht, The Netherlands; 2 Department of Vascular Surgery, University Medical Center Utrecht, Utrecht, The Netherlands

**Keywords:** Blunt thoracic aortic injury, Blunt traumatic aortic injury, Thoracic endovascular aortic repair, Left subclavian artery

## Abstract

**Purpose:**

Treatment of blunt thoracic aortic injuries (BTAIs) has shifted from the open surgical approach to the use of thoracic endovascular aortic repair (TEVAR), of which early outcomes appear promising but controversy regarding long-term outcomes remains. The goal of this study was to determine the long-term TEVAR outcomes for BTAI, particularly radiographic outcomes, complications and health-related quality of life (HRQoL).

**Methods:**

Retrospectively, all patients with BTAIs presented at a single level 1 trauma center between January 2008 and December 2018 were included. Radiographic and clinical outcomes were determined (early and long term). In addition, HRQoL scores using EuroQOL-5-Dimensions-3-Level (EQ-5D-3L) and Visual Analog Scale (EQ-VAS) questionnaires were assessed, and compared to an age-adjusted reference and trauma population.

**Results:**

Thirty-one BTAI patients met the inclusion criteria. Of these, 19/31 received TEVAR of which three died in hospital due to aorta-unrelated causes. In total, 10/31 patients died due to severe (associated) injuries before TEVAR could be attempted. The remaining 2/31 had BTAIs that did not require TEVAR. Stent graft implantation was successful in all 19 patients (100%). At a median radiographic follow-up of 3 years, no stent graft-related problems (endoleaks/fractures) were observed. However, one patient experienced acute stent graft occlusion approximately 2 years after TEVAR, successfully treated with open repair. Twelve patients required complete stent graft coverage of the left subclavian artery (LSCA) (63%), which did not result in ischemic complaints or re-interventions. Of fourteen surviving TEVAR patients, ten were available for questionnaire follow-up (follow-up rate 71%). At a median follow-up of 5.7 years, significant HRQoL impairment was found (*p* < 0.01).

**Conclusion:**

This study shows good long(er)-term radiographic outcomes of TEVAR for BTAIs. LSCA coverage did not result in complications. Patients experienced HRQoL impairment and were unable to return to an age-adjusted level of daily-life functioning, presumably due to concomitant orthopedic and neurological injuries.

**Electronic supplementary material:**

The online version of this article (10.1007/s00068-020-01432-y) contains supplementary material, which is available to authorized users.

## Introduction

Blunt thoracic aortic injury (BTAI) is a potentially lethal consequence of high-energy blunt thoracic trauma. Occurring in approximately 1% of motor vehicle crashes, merely an estimated 20% of BTAI patients will arrive at the hospital alive [1, 2]. Due to the relative mobility of the aortic arch and a fixed descending aorta, injuries to the thoracic aorta are usually located around the isthmus, just distal to the left subclavian artery (LSCA) [3, 4]. According to the Azizzadeh et al. grading scale, intimal irregularities of the thoracic aorta can be classified as grade I injuries, intramural hematomas as grade II, pseudoaneurysms as grade III and ruptures with free extravasation as grade IV [5].

Over the past decades, (surgical) treatment of BTAI changed significantly, shifting from the traditional open repair (aortic clamping and cardiopulmonary bypass) to endovascular repair, and making use of hybrid techniques in the case of associated injuries [6–8]. The use of thoracic endovascular aortic repair (TEVAR) for BTAI is a relatively novel development and in polytrauma patients usually performed in an emergency setting. Due to this emergency setting that limits pre-operative planning and BTAI morphology that often requires LSCA coverage for a sufficient proximal stent graft landing zone, outcomes may be different compared to other TEVAR procedures.

Timing of aortic repair remains challenging. Predominantly described in retrospective cohort studies, timing of repair depends on the severity of the BTAI, the total trauma burden and comorbidities [9–11]. The management strategy most often described in literature states that patients with significant aortic injuries (e.g., grade II, III and IV) in the absence of severe concomitant injuries should receive early TEVAR. Patients with severe associated injuries can be treated by delayed aortic repair, preceded by balanced resuscitation, antihypertensive therapy and impulse control while managing associated injuries before aortic repair is attempted [12–17].

Long-term outcomes regarding patency and complications of TEVAR for BTAI remain largely unknown [18–20]. Health-related quality of life (HRQoL) of BTAI survivors who often suffer from severe concomitant injuries is not determined yet. Therefore, their potential to return to normal daily-life function remains unknown.

This study displays our 10-year institutional experience with BTAI, radiological outcomes and complications after TEVAR and HRQoL at long-term follow-up. In addition, as our institution has adopted a BTAI treatment strategy that advocates early aortic repair and is predominantly dictated by hemodynamic parameters and presence of associated injuries, institutional BTAI management is depicted.

## Methods

### Setting

This retrospective cohort study was conducted at a Dutch level 1 trauma center that provides 24-h emergency care for trauma and vascular surgery, and has hybrid operating techniques and interventional radiology services available 24/7.

### Patient selection

All patients suffering from high-energy blunt thoracic trauma resulting in aortic injury, admitted to our hospital, between January 2008 and December 2018 were identified and included in this study. Patients were identified by searching the prospectively collected institutional trauma database, according to the corresponding Abbreviated Injury Scale (AIS) and the International Classification of Diseases (ICD) 10 codes for thoracic aortic injuries. Patients < 18 years old at the time of injury were excluded. The Azizzadeh et al. grading scale was used to grade the severity of BTAIs [5].

### Patient characteristics

Baseline characteristics and additional patient data such as injury severity score (ISS), mechanism of injury, aortic injury grading and (endovascular) treatment specifications, were extracted from medical reports and the trauma database. Polytrauma was defined as an ISS ≥ 16. Hemodynamic instability was defined as severe shock with the need for (massive) transfusion and/or vasopressor support without evident response, as decided by the treating surgeon. All other patients were considered hemodynamically stable (enough) to undergo computed tomography angiography (CTA) scanning. Thoracic injuries were defined as all intra-thoracic soft-tissue injuries, with or without fractures of the sternum, ribs, scapula and clavicles, excluding the aortic injury. Abdominal injuries were defined as all soft-tissue injuries to the abdomen. Cervical, thoracic and lumbar spine injuries were defined as all spine fractures, dislocations and discoligamentous injuries. Traumatic brain injury (TBI) was defined as all traumatic intra-cranial injuries such as severe intra-cranial swelling, hemorrhage, contusions and diffuse axonal injuries (visible on CTA scan or (delayed) magnetic resonance imaging (MRI) scan). Spinal cord injury was defined as both complete and incomplete injuries to the spinal cord. Associated injuries were (accurately) determined and noted in the records of all patients who received (full) body CTA-scanning. If patients died prior to CTA-scanning, only the encountered injuries could be noted. LSCA coverage by TEVAR was defined as complete/full coverage, leading to greatly reduced/absent antegrade flow in the LSCA, visible during procedural angiography and post-operative CTA-scanning. Procedural mean arterial pressure (MAP) was based on the continuous measurements of vital parameters during TEVAR.

### Diagnostics

**Fig. 1:**
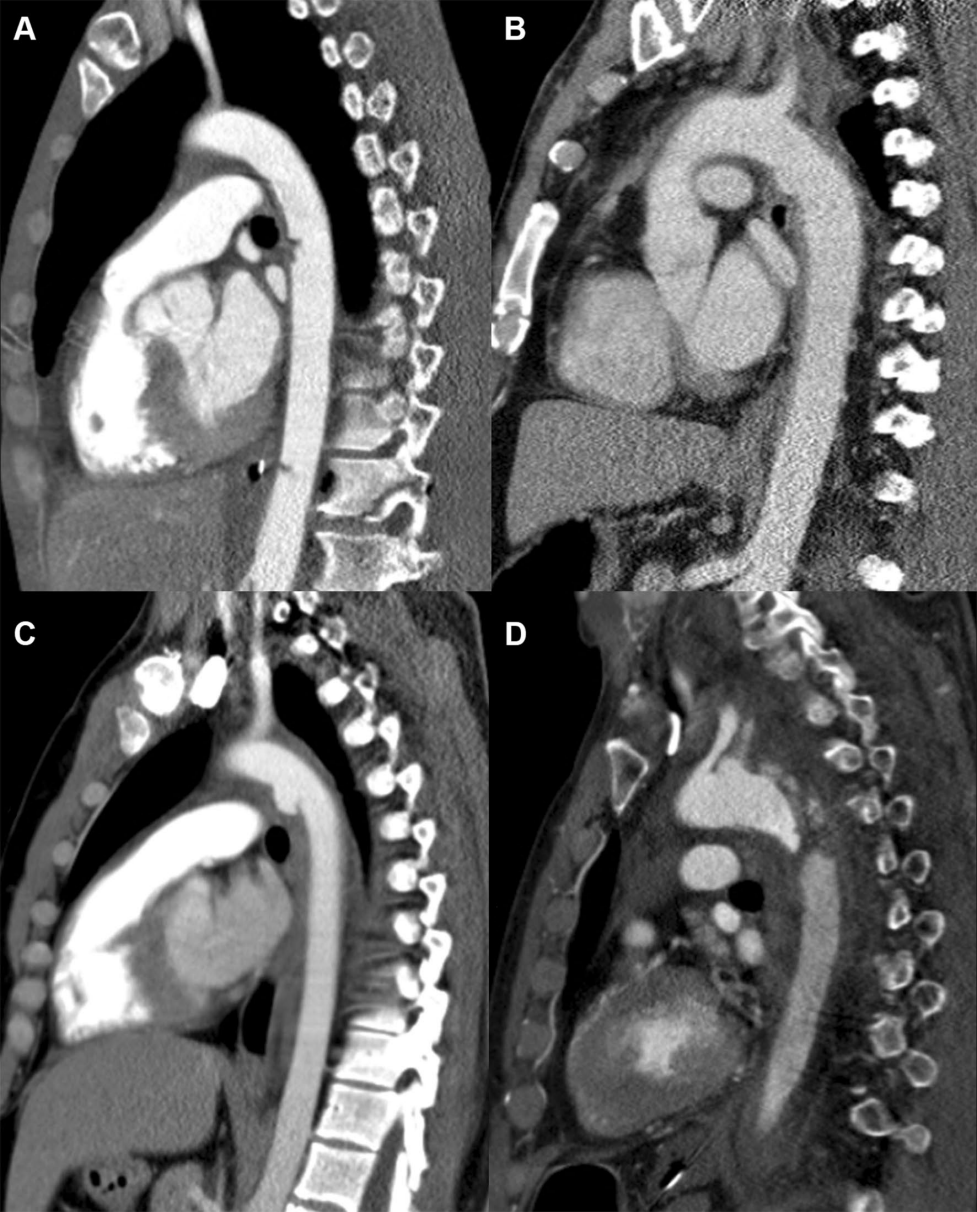
Computed tomography angiography images of the four Azizzadeh et al. blunt thoracic aortic injury (BTAI) grades. **a** Grade I, intimal tear: two intimal tears of the descending thoracic aorta are visible. **b** Grade II, intramural hematoma: distal side of the aortic arch. **c** Grade III, pseudoaneurysm: distal to the left subclavian artery. **d** Grade IV, rupture: aortic arch disruption causing massive hemothorax resulting in decreased contrast enhancement of the descending aorta

**Fig. 2:**
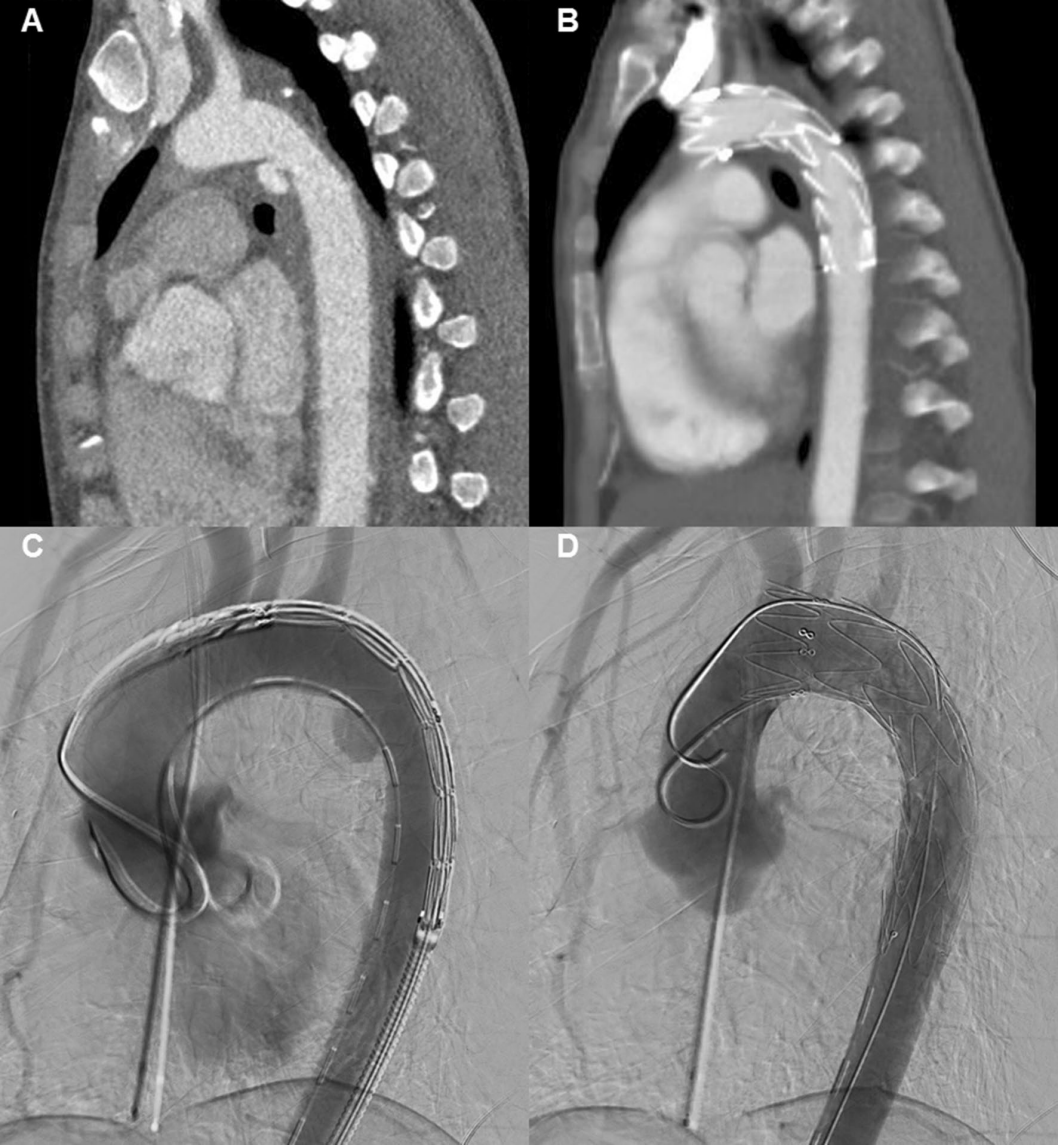
**a** Pre-operative CTA scan of the thoracic aorta, displaying a grade III injury (pseudoaneurysm, without extravasation) located near the isthmus. **b** Post-operative CTA scan, displaying pseudoaneurysm coverage by TEVAR. **c** Procedural angiography displaying the aortic pseudoaneurysm before stent graft deployment. **d** Procedural angiography after proximal bare stent graft deployment, covering the pseudoaneurysm and left subclavian artery (visible due to retrograde flow)

The initial diagnosis of BTAI was made by the attending radiology consultant, in the case of patients who underwent CTA-scanning. Grading of BTAIs was based on the injury description provided in the radiology records, using the Azizzadeh et al. grading scale (Fig. 1). Uncertainties on the severity of BTAIs were resolved after review of CTA-scans and procedural angiography images by radiology, trauma- and vascular surgery consultants (Fig. 2). Patients with grade IV BTAIs (free ruptures) who were hemodynamically unstable, and therefore could not undergo initial CTA-scanning and did not survive were graded based on operative and chest X-ray findings.

### Outcomes and follow-up

Long-term (radiographic) follow-up was achieved by following the surviving patients with BTAI in an outpatient setting. Imaging of the thoracic aorta was performed at 1-, 2- and 5-year intervals, and was reviewed by both radiologists and vascular surgeons. In addition, in-hospital mortality and thrombo-embolic/ischemic complications of BTAI patients were determined. The National Personal Records Database was queried to determine post-discharge mortality.

Health-related quality of life was determined using the EuroQOL 5-Dimensions 3-Level (EQ-5D-3L) questionnaire. The EQ-5D covers five dimensions (mobility, self-care, usual activities, pain/discomfort and anxiety/depression) that are all divided into three levels: no problems, moderate problems, or severe problems. The EQ-5D index scores were calculated using the Dutch tariff [21]. In addition, the EuroQOL Visual Analog Scale (EQ-VAS) was assessed. This score represents a patient’s self-rated health status on a scale from 0 to 100, a score of 0 being the worst imaginable health state and a score of 100 being the best imaginable health state. The EQ-5D age-adjusted population index scores for the Netherlands and the EQ-5D index score of the general trauma population admitted to the same institution as the study population determined by Van der Vliet et al. were used to compare scores in TEVAR patients [21, 22].

### Institutional management strategy

Institutional policy regarding BTAI is primarily based on hemodynamic stability. Stable patients undergo (full-body) CTA-scanning, profoundly unstable patients (non-responders) suspicious for severe thoracic or abdominal bleeding (based on chest X-ray or extended focused assessment with sonography for trauma (eFAST) findings), generally undergo immediate thoracotomy or laparotomy, respectively. Unstable patients with a (limited) response to resuscitation will mostly undergo full-body CTA-scanning, if deemed feasible by the treating surgeon. After emergency surgery and resuscitation, these patients receive (full body) CTA-scanning. In the case of BTAI (irrespective of BTAI grading) with severe concomitant injuries (e.g., active intra-cranial or abdominal bleeding, pulmonary contusions, complex extremity fractures), patients undergo simultaneous treatment for BTAI and (life-threatening) associated injuries, performing TEVAR and using hybrid techniques. The rationale behind this strategy is that these patients, for instance suffering from severe TBI, will presumably not tolerate antihypertensive therapy and impulse control needed in case of delayed TEVAR. In addition, in case of hemodynamic instability that is (partially) attributable to BTAI, TEVAR is performed to optimize bleeding control and prevent diffuse ischemic complications and secondary shock. In contrast, patients suffering from BTAI with relative minor associated injuries will receive antihypertensive therapy, impulse control and delayed TEVAR in a semi-acute setting (< 48 h). Delayed TEVAR was defined as the aortic repair performed in a semi-acute setting after antihypertensive therapy and impulse control were initiated, or after other surgical procedures were performed and normal physiology was restored (irrespective of delay duration).

### Statistical analysis

Statistical and descriptive analyses of patient baseline characteristics demographics, outcomes and questionnaire results are performed using IBM SPSS Statistics version 24 (IBM Corp., Armonk, NY). Continuous variables are reported as means with standard deviations (SD) and medians with interquartile ranges (IQR). Categorical data are displayed as numbers with percentages. The EQ-5D index scores are compared with the Dutch reference population index norm of the same age using two-sample *t* test [21]. Categorical variables are analyzed using Chi square test. The relation between continuous outcome measures and dichotomous explanatory variables is assessed using the Mann–Whitney *U* test. A *p*-value of < 0.05 is considered significant.

## Results

### Patient demographics and institutional management

**Fig. 3:**
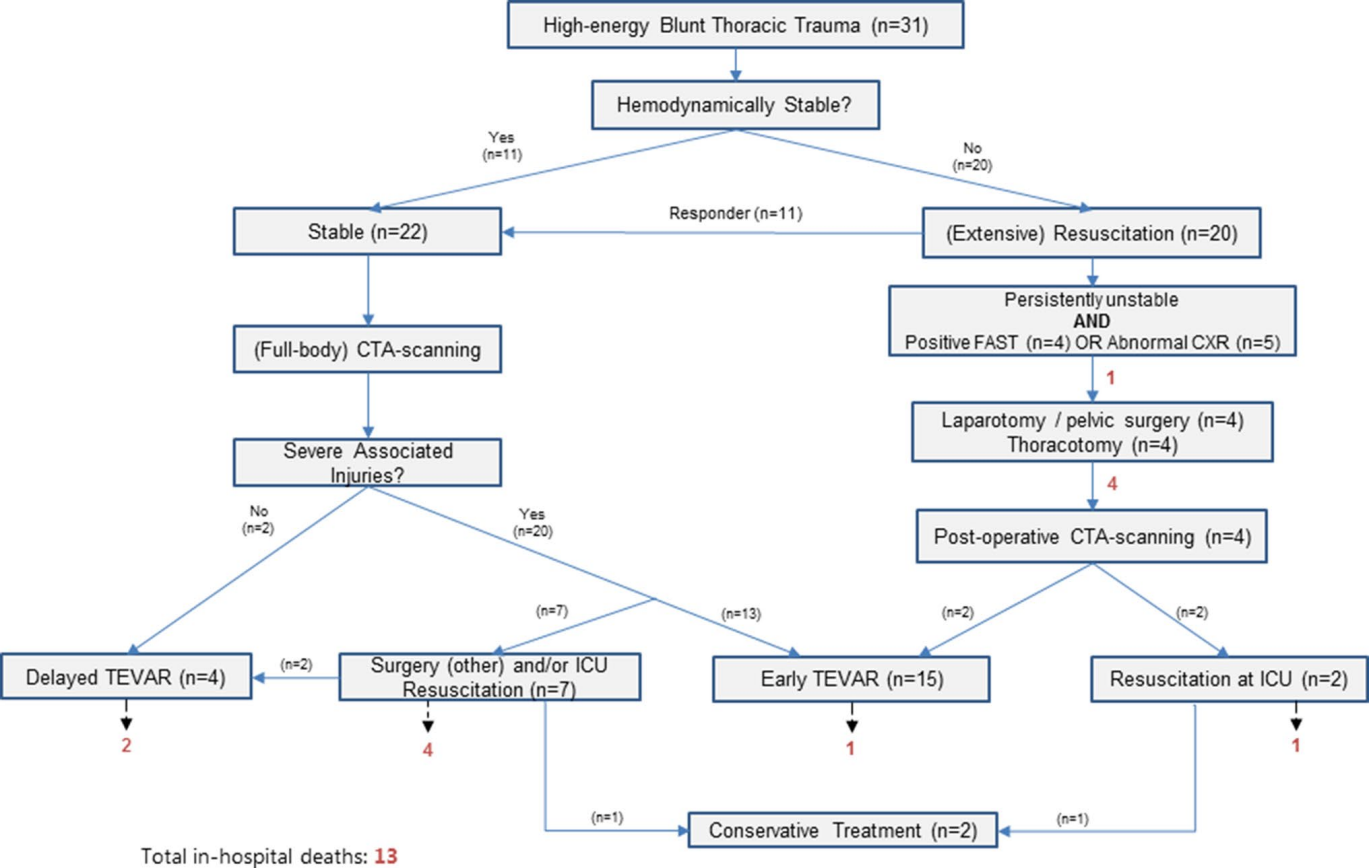
Flowchart displaying institutional management of blunt thoracic aortic injury. Number of in-hospital mortalities are depicted as red colored numbers. *TEVAR* thoracic endovascular aortic repair, *FAST* focused assessment with sonography in trauma, *CXR* chest X-ray, *CTA* computed tomography angiogram, *ICU* intensive care unit. Patients were considered hemodynamically unstable if they had no sufficient response to massive transfusion. Other patients were stable (enough) and underwent CTA-scanning

A total of 31 BTAI patients were identified. Baseline characteristics of these patients and the 19 of them who received TEVAR are shown in Table 1. Median ISS was 38, all were polytrauma patients. Motor vehicle collisions were the responsible trauma mechanism in 25/31 cases (81%). Of the 31 BTAI patients, ten patients died due to severe associated injuries before definitive aortic repair was attempted, of which none were able to receive open repair. Causes for in-hospital mortality are depicted in Table 1. Nineteen patients received TEVAR, of which 18 patients suffered from grade III BTAIs (pseudoaneurysms without active extravasation). Patient and treatment characteristics stratified for aortic injury grading are displayed in supplementary material Table 1. In 15/19 patients, early TEVAR was performed due to their extensive associated injuries, which are displayed in Table 2. The other four patients received delayed TEVAR, with a delay ranging from 0.25 to 0.50 days. Three of the 19 patients in the TEVAR group died in hospital (16%), all due to BTAI-unrelated causes, as it is depicted in Table 1. Two patients received conservative treatment regarding their BTAI, of which one died seven months after hospital discharge for which no cause could be determined. Institutional management of BTAI, including observed in-hospital mortalities, is displayed using a flowchart (Fig. 3).

**Table 1:** Characteristics of all BTAI patients and of those who received TEVAR

	All BTAI patients (*n* = 31)	TEVAR patients (*n* = 19)
Age (years)	33 [23–48]	35 [25–46]
Male	24 (77)	16 (84)
ISS	38 [29–54]	34 [29–50]
Polytrauma	31 (100)	19 (100)
Mechanism of injury		
MVC	25 (81)	17 (90)
Fall from height (> 2 m)	3 (10)	1 (5)
Pedestrian collision	1 (3)	0 (0)
Blast injury	1 (3)	0 (0)
Train collision	1 (3)	1 (5)
Systolic blood pressure	100 [80–120]	110 [80–120]
≤ 90 mmHg	15 (48)	6 (32)
Pulse (bpm)	100 [85–120]	103 [85–120]
Hemodynamic instability	9 (29)	2 (11)
Cardiac arrest upon arrival at the ED	5 (16)	0 (0)
Hb (mmol/L)	7.8 [6.9–8.7]	8.0 [7.0–8.9]
Arterial pH^a^	7.26 [7.09–7.32]	7.26 [7.13–7.33]
Lactate (mmol/L)^a^	3.3 [2.5–5.6]	3.2 [2.5–5.6]
Base deficit (mEq/L)^a^	7.0 [3.1–11.0]	5.0 [3.0–10.0]
BTAI injury grade^b^		
Grade I	1 (3)	0 (0)
Grade II	2 (6)	1 (5)
Grade III	22 (71)	18 (95)
Grade IV	6 (19)	0 (0)
Treatment (aortic repair)		
Early TEVAR	15 (48)	15 (79)
Delayed TEVAR	4 (13)	4 (21)
Conservative	2 (6)	N/a
Died before repair	10 (32)	N/a
Additional treatment^c^		
Craniotomy	1 (3)	1 (5)
Thoracotomy	4 (13)	0 (0)
Laparotomy	7 (23)	5 (26)
Pelvic fracture surgery	3 (10)	1 (5)
Spinal fracture surgery	5 (16)	3 (16)
Extremity fracture surgery	8 (26)	6 (32)
Hospital stay (days)	10 [0–26]	21 [8–41]
ICU stay (days)	2 [0–11]	9 [1–13]
Outcomes		
In-hospital mortality	13 (42)	3 (16)
BTAI-related mortality	5 (16)	0 (0)
Cause of in-hospital death		
Hemorrhage (aortic)	4 (13)	0 (0)
Multi-organ failure	3 (10)	1 (5)
Traumatic brain injury	3 (10)	2 (11)
Myocardial contusion	2 (6)	0 (0)
Futility	1 (3)	0 (0)

Data are presented as the number (%) or the median [IQR: 25th–75th percentile]

*TEVAR* thoracic endovascular aortic repair, *BTAI* blunt thoracic aortic injury, *MVC* motor vehicle collision, *ISS* injury severity score, *ICU* intensive care unit, *Hb* hemoglobin

^a^Arterial blood gas results were missing in 3 non-TEVAR patients

^b^According to Azizzadeh et al. grading scale

^c^Multiple additional procedures were performed in multiple patients

**Table 2:** Associated injuries and procedure details of patients who received TEVAR

	TEVAR patients (*n* = 19)
Associated injuries^a^	
Brain injury	10 (53)
Thoracic injury	17 (90)
Abdominal injury	11 (58)
Pelvic injury	8 (42)
Extremity fracture	8 (42)
Associated spine injuries^a^	
Cervical	5 (26)
Thoracic	5 (26)
Lumbar	5 (26)
Spinal cord injury	3 (16)
TEVAR procedure details	
TEVAR procedure time (minutes)	95 [80–132]
Median (IQR) TEVAR diameter (mm)	26 [24–30]
Median (IQR) TEVAR length (mm)	150 [100–151]
Median (IQR) procedural MAP (mmHg)	68 [62–79]
Simultaneous surgery patients^b^	6 (32)
Laparotomy	3 (16)
Extremity surgery	2 (11)
Craniotomy	1 (5)
Spine fracture surgery	1 (5)
Additional procedure time (minutes)	75 [20–153]
TEVAR procedure time (minutes)	115 [80–125]

Data are presented as the number (%)

*TEVAR* thoracic endovascular aortic repair, *MAP* mean arterial pressure

^a^Multiple injuries were found in multiple patients

^b^Multiple surgeries were performed in multiple patients, reported TEVAR procedure time is for patients undergoing simultaneous surgery (utilizing hybrid techniques)

### Procedure details

Of the 19 patients who received TEVAR, data regarding used stent grafts (including dimensions) were available in 18 cases (95%). All patients were treated with stent grafts with a proximal bare stent. In the period 2008–2010, we used the Relay^®^ Thoracic Stent Graft (Bolton Medical, Sunrise, Florida, USA; *n* = 4) and the Valiant™ Thoracic Stent Graft (Medtronic, Dublin, Ireland; *n* = 1). Between 2011 and 2018, we used the Valiant™ Thoracic Stent Graft (Medtronic, Dublin, Ireland; *n* = 13). In 6 out of 15 patients (40%) who received early TEVAR, treatment for associated injuries was performed in the same operating session (utilizing hybrid techniques). In these six patients, three laparotomies, two extremity surgeries, one craniotomy and one spinal fracture surgery were performed. TEVAR procedure details, including operating times, stent graft sizing and procedural blood pressure measurements are displayed in Table 2.

### Early outcomes

Early outcomes of the 19 patients who received TEVAR are displayed in Table 3. All patients had successful TEVAR stent graft implantation, without any conversions to open aortic surgery and no significant signs for endoleaks on all in-hospital post-operative CTA scans. In one case, TEVAR implantation caused a left-sided iatrogenic common carotid artery pseudoaneurysm which did not require an extra intervention. In 12/19 patients, aortic dissection morphology required complete LSCA coverage (63%). This did not result in ischemic complaints of the left arm. Four out of 19 TEVAR patients were found to have brain ischemia/ischemic stroke during hospitalization (21%), of which all had LSCA coverage and concomitant severe TBI (impairing cerebral circulation). Of these four cases of cerebral ischemia, one had no evidence of a posterior or anterior stroke, the second suffered from diffuse ischemia due to brain herniation as a result of severe intra-cranial bleeding, the third had anterior ischemic stroke caused by an ipsilateral traumatic carotid artery dissection, whereas the last patient, the one suffering from an iatrogenic carotid artery pseudoaneurysm, had bilateral watershed cerebral ischemia that implied a hemodynamic cause. In all of these cases, LSCA coverage was deemed an unlikely source of cerebral ischemia. Postoperatively, one patient suffered from a partial spinal cord injury, which did not significantly improve during hospital stay. This was most likely caused by a traumatic contusion of the spinal cord (in the absence of vertebral fractures) or hemodynamic instability due to trauma with reduced spinal cord perfusion as consequence. Diffusion-weighted MRI was inconclusive for the origin of the spinal cord injury, due to TEVAR artifacts projecting over the site of injury.

**Table 3:** Early outcomes of patients undergoing TEVAR (*n* = 19) and long-term (radiographic) outcomes (*n* = 16)

Early outcomes	(*n* = %)	Long-term outcomes	(*n* = %)
Implantation success	19 (100)	BTAI-related out-of-hospital mortality	0 (0)
Complete left subclavian artery coverage	12 (63)	Patients lost to follow-up^c^	3 (19)
Median (IQR) delay (days)^a^	0.4 (0.25–0.50)	Median (IQR) radiographic follow-up (years)	3.0 (2.1–5.5)
Cerebral ischemia	4 (21)	Endoleaks	0 (0)
Access route injury	1 (5)	Stent fracture	0 (0)
Conversion	0 (0)	Occlusion and need for open repair	1 (6)
Left arm ischemia	0 (0)	Left arm claudication	0 (0)
Spinal cord ischemia^b^	1 (5)		
Endoleaks	0 (0)		
In-hospital mortality	3 (16)		
BTAI-related in-hospital mortality	0 (0)		

*TEVAR* thoracic endovascular aortic repair

^a^Only 4 patients received delayed aortic repair

^b^Multiple causes for spinal cord ischemia were identified, see results/early outcomes

^c^One patient died in out-of-hospital palliative care institution, one patient refused follow-up imaging and outpatient consults, one patient is yet to receive radiographic follow-up

### Traumatic brain injury

In total, 10/19 TEVAR patients suffered from (severe) TBI (53%), consisting of cerebral contusions (*n* = 4), intra-cranial bleeding (*n* = 2), diffuse axonal injury (*n* = 2), cerebral ischemia (*n* = 1) and diffuse cerebral swelling (*n* = 1). Median MAP during TEVAR in patients with TBI was 69 mmHg (67–80) and in those without TBI 61 mmHg (59–73), showing a trend towards a higher MAP in those with TBI (*p* = 0.06). In 4/10 cases progression of TBI was visible on follow-up imaging (40%). One of these had severe intra-cranial bleeding, cerebral herniation and diffuse ischemia, resulting in death after discharge to a palliative care institution. Two had diffuse axonal injuries, of which the one with additional brain stem lesions died in due course. The last patient deceased from severe TBI had multiple cerebral ischemic foci after losing cardiac output on scene. In patients with TBI progression, median procedural MAP was 74 mmHg (67–80) and in those without progression 69 mmHg (66–79), which was not significantly different (*p* = 0.71).

### Long-term outcomes

Long-term outcomes of TEVAR patients are described in Table 3. Three patients died due to aorta-unrelated causes. Median (IQR) radiographic follow-up was 3.0 years (2.1–5.5 years), mean radiographic follow-up was 3.7 years. In 13/16 patients, radiographic follow-up was available. Of the three patients who did not receive radiographic follow-up, one refused follow-up, the second died due to BTAI-unrelated causes before imaging was performed and the last is yet to receive radiographic follow-up. In one patient, acute occlusion of TEVAR stent graft occurred approximately 1.9 years after discharge, resulting in emergent open aortic repair which the patient survived. At the time of occlusion, the patient was on antiplatelet therapy (Carbasalate calcium, 100 mg daily). No evidence for thrombophilia was found; however, a combination of anatomical factors (steep angulated aortic arch) in combination with stent graft-related factors (structural stiffness) may have played a role in the occurrence of this complication [23]. No other significant radiographic complications or patency issues, particularly stent fractures or endoleaks, were identified during the follow-up period.

### Health-related quality of life

**Fig. 4:**
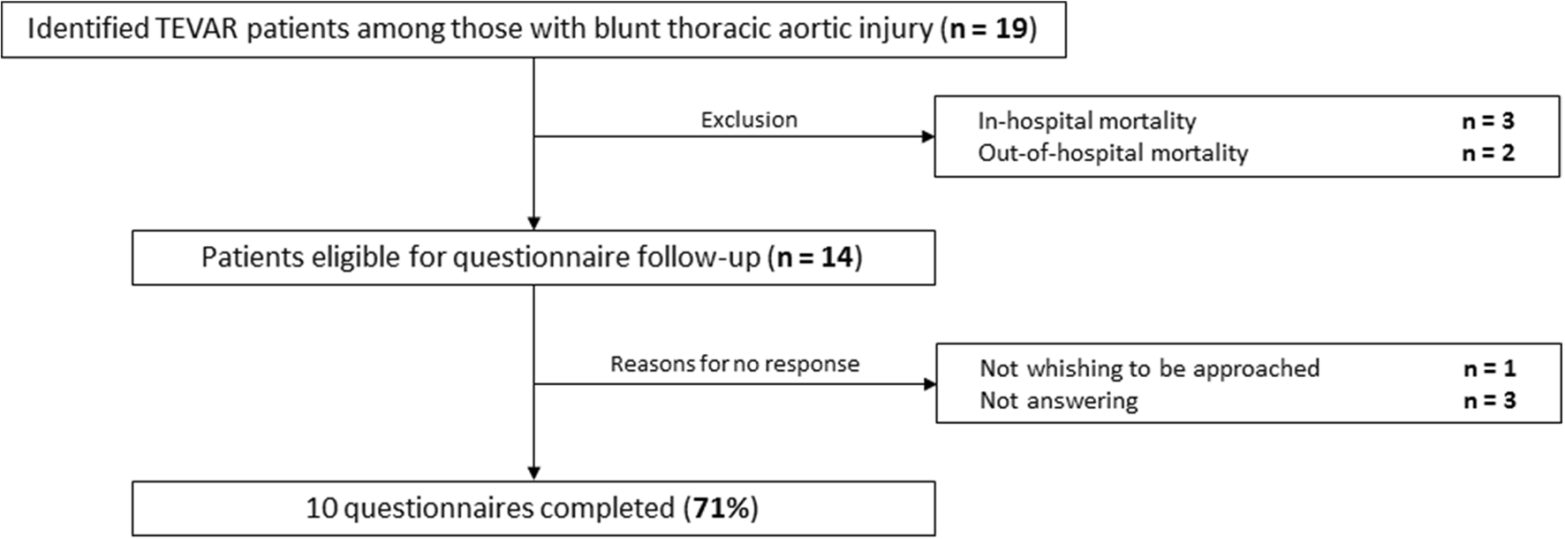
Flowchart displaying exclusions and responses to EQ-5D questionnaire follow-up

**Fig. 5:**
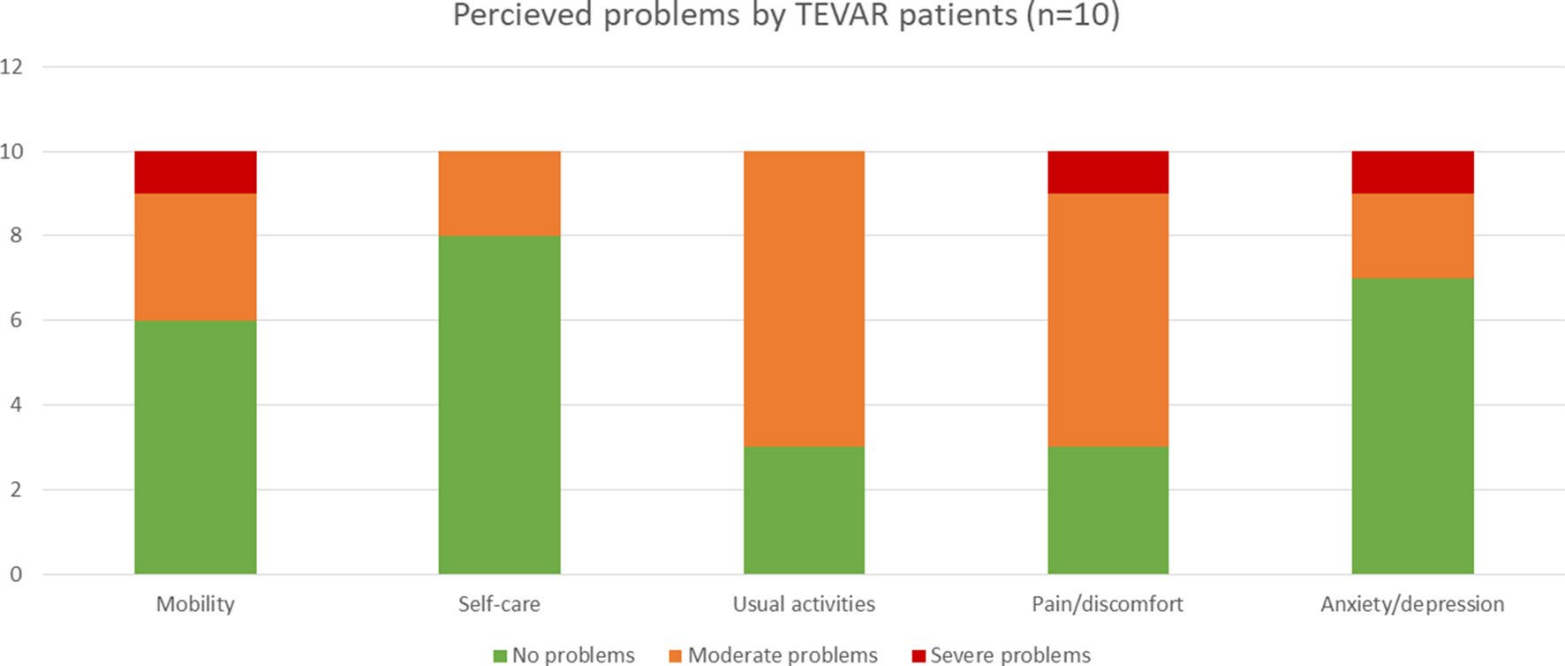
EQ-5D-3L questionnaire reported problems of the TEVAR patients available at long-term follow-up

We were able to determine EQ-5D results in 10 patients (response rate 71%) (Fig. 4). Median age at completion of the questionnaires was 41 (30–51) years. Median (IQR) time from discharge to questionnaire follow-up was 5.7 (2.2–8.5) years. The median (IQR) and mean (SD) EQ-5D index scores for TEVAR patients were 0.75 (0.53–0.92) and 0.69 (0.31) respectively, which was significantly lower in comparison to the general Dutch population of the same age (*p* < 0.01), but not to a general multitrauma population (*p* = 0.61) as is depicted in Table 4 [22]. The median (IQR) and mean (SD) EQ-VAS scores were 70 (58–78) and 68 (25), respectively, significantly lower than the EQ-VAS score of the Dutch population of the same age (*p* = 0.04) [21]. Domains in which patients experienced most problems were performance of usual activities and pain/discomfort, as displayed in Fig. 5.

**Table 4:** Mean (SD) EQ-5D index scores compared to reference populations

	Reference	TEVAR patients	*P*-value
Dutch population (age 35–45)^a^	0.94 (0.18)	0.69 (0.31)	< 0.01
General trauma population UMCU^b^	0.74 (0.31)	0.69 (0.31)	0.61

*TEVAR* thoracic endovascular aortic repair, *UMCU* University Medical Center Utrecht

^a^Derived from Janssen et al. [21]

^b^Derived from Van der Vliet et al. [22]

## Discussion

BTAI patients who were able to receive TEVAR did survive their aortic injury, and those who survived their concomitant injuries had acceptable early and long-term outcomes. During the three-year radiographic follow-up, no significant signs of endoleaks or stent failure were observed.

### Left subclavian artery coverage

These relatively young trauma patients who require TEVAR are at risk for the type 1 endoleaks and re-interventions, because of their highly angulated aortic arch, short distance between the aortic injury and the LSCA and potential dilatation of the aorta at long-term follow-up [24, 25]. Therefore, stent graft coverage of the LSCA is often indicated. In our cohort, none of the patients with complete LSCA coverage developed ischemic complaints of the left arm (both at short- and long-term follow-up), and no significant endoleaks were observed. We did find that 4/12 patients who received complete LSCA coverage had signs of cerebral ischemia/ischemic stroke visible at CTA-scanning. However, all were rather most likely the results of (severe) TBI. Discussion remains whether LSCA coverage increases the risk of ischemic stroke [26]. It is proposed that LSCA coverage, and therefore unilateral vertebrobasilar insufficiency, may increase the chance of posterior ischemic stroke and not of anterior ischemic stroke [27, 28]. We did not find evident ischemic strokes of the posterior circulation that could be related to LSCA coverage, which is in contrast to what has been suggested in literature [27, 28]. Based on the results found in our population, we speculate that complete LSCA coverage during TEVAR is relatively safe and did not lead to ischemic complications or re-interventions.

### Health-related quality of life

BTAI patients who received TEVAR reported significantly diminished HRQoL compared to the general Dutch population of the same age at long-term follow-up. When comparing TEVAR patients to the admitted general trauma population at the same institution, we did not find a difference in HRQoL impairment at long-term follow-up. However, it must be noted that TEVAR patients had a median age of 41 years at follow-up; while, the general trauma population reported by Van der Vliet et al. had a median age of 55 years old [22]. As EQ-5D scores are age dependent, with scores decreasing with increasing age, it is possible that TEVAR patients would have lower index scores (experiencing more impairment) at a later follow-up moment [21]. When looking at domains of HRQoL impairment, most patients experienced problems while performing their daily activities and reported pain/discomfort. We speculate that these impairments may be related to concomitant injuries. Since no patients experienced ischemic complaints of their left arm or any other stent graft-related problems, it appears that the BTAI itself plays a smaller role in the cause of HRQoL impairment at long-term follow-up. This can be supported by the finding that no difference between EQ-5D index scores of TEVAR patients and that of the admitted general trauma population was observed.

### Institutional management and timing of TEVAR

We depicted institutional BTAI management and the early TEVAR strategy (utilizing hybrid techniques if applicable) using a flowchart, to illustrate decision-making after high-energy blunt thoracic trauma based on hemodynamic stability (Fig. 3). Hemodynamically stable patients receive (whole body) CTA-scanning. Unstable patients with a (limited) response to resuscitation will receive (whole body) CTA-scanning if deemed feasible by the treating surgeon. This is in accordance with the current literature regarding the role of CT-scanning in thoracic trauma patients, advocating a more liberal use of whole body CT-scanning after thoracic trauma [29]. However, this is highly dependent on institutional facilities, resources and logistics. Even more, profoundly unstable patients, unresponsive to resuscitation with an identified potential source of blood loss, receive immediate (life-saving) surgery. Of all patients who were deemed hemodynamically stable and could undergo CTA-scanning, and unstable patients with a response to resuscitation where CTA-scanning was deemed feasible, none of the patients who received early TEVAR died. All mortalities that were observed in patients who received (delayed) TEVAR were not aortic injury related. Based on these results, we conclude that an early aortic repair strategy in patients who are able to receive TEVAR does not lead to aorta-related in-hospital mortality.

In our study, more than half of the TEVAR patients suffered from (severe) TBI. Of these, four patients who had severe concomitant TBI showed progression of their brain injuries (40%), of which progression in all cases was most likely related to the extent of the initial brain injury rather than procedural hypotension.

The study of Rabin et al. that specifically investigated TBI progression in BTAI patients who received early versus delayed aortic repair, reports progression of TBI in patients who received early aortic repair for BTAI [30]. Delayed BTAI repair in patients with TBI, if allowed by the type of aortic injury, was proposed. Although the majority of early aortic repair patients in the Rabin et al. study received open repair (requiring cardio-pulmonary bypass) and the majority of delayed repair patients received TEVAR, early repair was reported to be independently associated with TBI progression. However, since procedural details such as MAP were not reported, and heterogeneity of injuries and treatment modalities was present, we speculate that treatment-related differences in the early and delayed group still may have affected results.

It is hypothesized that procedural hypotension during early aortic repair in patients with TBI may cause progression of intra-cranial injuries. Procedural hypotension is necessary to minimize stent graft movement and promote the accuracy of deployment, which can be achieved by administering short-acting vasodilators or rapid pacing, of which the latter may be preferred due to the very short duration of hypotensive periods with almost instantaneous recovery [31]. Although our patient sample is relatively small, we did not find a difference in procedural MAP in patients with and those without progression of TBI. We, therefore, speculate that the observed TBI progression was a result of the natural progression of the severe initial injury to the brain rather than the result of an early TEVAR strategy. However, as ‘delayed’ TEVAR patients at our institution received stent graft implantation in a semi-acute setting (without more than a day of delay), we were not able to compare TBI progression of early TEVAR to an actual delayed TEVAR group. In addition, we hypothesize that (although infrequent) a secondary rupture of (grade 3) BTAIs before delayed TEVAR is performed, has the potential to cause severe cerebral ischemia and can be prevented by early aortic repair. Larger studies focusing on complications caused by early and delayed BTAI repair (strictly using TEVAR) are necessary to determine the potential benefits and safety of the early repair strategy.

### Limitations

Our study has several limitations. This was a retrospective study with a small number of patients. As our results indicate promising results of TEVAR for BTAI, specifically the safety and effectiveness of LSCA coverage, larger (prospective) studies are necessary to determine if stent graft-related complications could be similar to the ones we found. More importantly, future studies should indicate if device-related complication remain absent during a longer follow-up period. Furthermore, since the number of patients with severe concomitant injuries who received early TEVAR at our institution was small, larger studies are required to determine the safety and effectiveness of this strategy.

## Conclusions

This study shows good long(er)-term radiographic outcomes of BTAI patients who received TEVAR, and the absence of ischemic complaints and re-interventions after necessary complete LSCA coverage. In-hospital mortality of TEVAR patients was not aortic injury related. Early TEVAR in patients with severe concomitant injuries did not appear to cause higher peri-operative mortality. However, BTAI patients who were treated by TEVAR successfully did experience significant HRQoL impairment after high-energy thoracic trauma. Research with larger BTAI populations and a longer follow-up period are necessary to determine any adverse events associated with stent graft implantation. Optimal institutional management strategies are subject to available resources. It should be investigated whether our early aortic repair strategy (with the utilization of hybrid techniques if applicable) is equally feasible and safe in trauma centers with other resources available.

## Electronic supplementary material

Below is the link to the electronic supplementary material.

Supplementary material 1 (DOCX 17 kb)

## Data Availability

We have not filed for permission to publish the study material.
