# Supplementary material for: Blunt thoracic aortic injury and TEVAR: long-term outcomes and health-related quality of life
Source: Eur J Trauma Emerg Surg. 2020 Jul 6;48(3):1961–73. doi: 10.1007/s00068-020-01432-y (PMC9192473; doi:10.1007/s00068-020-01432-y)
Supplement: Supplementary file 1 — Supplementary material 1 (DOCX 17 kb) [file 68_2020_1432_MOESM1_ESM.docx]

**Supplementary materials**

**Table 1.** Characteristics of all BTAI patients according to injury grading.

|  | **Grade I**  (n = 1) | **Grade II** (n = 2) | **Grade III** (n = 22) | **Grade IV** (n = 6) |
| --- | --- | --- | --- | --- |
| Age (years) | 56 | 34 | 33 [23 – 50] | 33 [20 – 51] |
| Male | 1 (100) | 2 (100) | 17 (77) | 4 (67) |
| ISS | 29 | 46 | 40 [29 – 51] | 38 [23 – 75] |
| Systolic blood pressure | 120 | 100 | 100 [78 – 120] | 50 [0 – 110] |
| ≤ 90 mmHg | 0 (0) | 1 (50) | 9 (41) | 5 (83) |
| Pulse (bpm) | 70 | 106 | 103 [85 – 120] | 17 [0 – 101] |
| Hemodynamic instability | 0 (0) | 1 (50) | 3 (14) | 5 (83) |
| Cardiac arrest upon arrival at the ED | 0 (0) | 0 (0) | 1 (5) | 4 (67) |
| Hb (mmol/L) | 9.3 | 6.6 | 7.8 [6.8 – 8.4] | 7.5 |
| Arterial pH | 7.38 | 7.29 | 7.24 [7.09 – 7.34] | 7.08 |
| Lactate (mmol/L) | 3.6 | 2.3 | 3.3 [2.7 – 6.1] | 6.0 |
| Base deficit (mEq/L) | 3.00 | 5.00 | 8.0 [2.8 – 11.0] | 16.0 |
| Treatment (aortic repair) |  |  |  |  |
| Early TEVAR | 0 (0) | 1 (50) | 14 (64) | 0 (0) |
| Delayed TEVAR | 0 (0) | 0 (0) | 4 (18) | 0 (0) |
| Conservative | 1 (0) | 1 (50) | 0 (0) | 0 (0) |
| Died before repair | 0 (0) | 0 (0) | 4 (18) | 6 (100) |
| Additional treatment^a^ |  |  |  |  |
| Craniotomy | 0 (0) | 0 (0) | 1 (5) | 0 (0) |
| Thoracotomy | 0 (0) | 0 (0) | 0 (0) | 4 (67) |
| Laparotomy | 0 (0) | 1 (50) | 5 (23) | 1 (17) |
| Pelvic fracture surgery | 0 (0) | 1 (50) | 1 (5) | 1 (17) |
| Spinal fracture surgery | 1 (100) | 1 (50) | 3 (14) | 0 (0) |
| Extremity fracture surgery | 0 (0) | 1 (50) | 6 (27) | 1 (17) |
| Hospital stay (days) | 21 | 33 | 14 [5 – 35] | 0 [0 – 0] |
| ICU stay (days) | 2 | 8 | 6 [0 - 12] | 0 [0 – 0] |
| Outcomes |  |  |  |  |
| In-hospital mortality | 0 (0) | 0 (0) | 7 (32) | 6 (100) |
| BTAI related mortality | 0 (0) | 0 (0) | 1 (5) | 4 (67) |

Data are presented as the number (%) or the median [IQR: 25th – 75th percentile].
TEVAR, Thoracic Endovascular Aortic Repair; BTAI, Blunt Thoracic Aortic Injury; MVC, motor vehicle collision;
ISS, Injury Severity Score; ICU, Intensive care unit; Hb, Hemoglobin ^a^ Multiple additional procedures were performed in multiple patients.
If values are displayed as median without IQR, missing observations prevented IQR calculations.
